# Cell-Free DNA–Based Multi-Cancer Early Detection Test in an Asymptomatic Screening Population (NHS-Galleri): Design of a Pragmatic, Prospective Randomised Controlled Trial

**DOI:** 10.3390/cancers14194818

**Published:** 2022-10-01

**Authors:** Richard D. Neal, Peter Johnson, Christina A. Clarke, Stephanie A. Hamilton, Nan Zhang, Harpal Kumar, Charles Swanton, Peter Sasieni

**Affiliations:** 1 Department of Health and Community Sciences, Faculty of Health and Life Sciences, University of Exeter, Exeter EX4 4PY, UK; 2 NHS England, London SE1 8UG, UK; 3 GRAIL, LLC, a Subsidiary of Illumina, Inc., Menlo Park, CA 94025, USA; 4 GRAIL, LLC, a Subsidiary of Illumina, Inc., London, WC1V 7HP, UK; 5 Cancer Research UK Lung Cancer Centre of Excellence, University College London Cancer Institute, London, WC1E 6DD, UK; 6 Cancer Evolution and Genome Instability Laboratory, Francis Crick Institute, London NW1 1AT, UK; 7 Comprehensive Cancer Centre, King’s College London, London WC2R 2LS, UK

**Keywords:** multi-cancer early detection, liquid biopsy, cancer screening, cell-free nucleic acids, population screening, randomised controlled trial, efficient design

## Abstract

**Simple Summary:**

NHS-Galleri is a randomised controlled trial that will assess how well a blood test can reduce the number of late-stage cancers by helping to find cancers early. The test looks at patterns in blood samples to detect a cancer signal. NHS-Galleri has enrolled over 140,000 people, invited from the general population of England aged 50–77 years who did not have or were not being investigated for cancer. Blood is being collected up to three times: first when people join the study and again 12 and 24 months later. Half the participants (chosen at random) will have their blood tested and half will have their blood stored. Participants will not be told whether their blood will be tested or stored. Participants with a cancer signal detected result will be sent for further diagnostic testing in the NHS. The trial will help the NHS decide whether to introduce screening using this test.

**Abstract:**

We report the design of the NHS-Galleri trial (ISRCTN91431511), aiming to establish whether a multi-cancer early detection (MCED) test that screens asymptomatic individuals for cancer can reduce late-stage cancer incidence. This randomised controlled trial has invited approximately 1.5 million persons and enrolled over 140,000 from the general population of England (50–77 years; ≥3 years without cancer diagnosis or treatment; not undergoing investigation for suspected cancer). Blood is being collected at up to three annual visits. Following baseline blood collection, participants are randomised 1:1 to the intervention (blood tested by MCED test) or control (blood stored) arm. Only participants in the intervention arm with a cancer signal detected have results returned and are referred for urgent investigations and potential treatment. Remaining participants in both arms stay blinded and return for their next visit. Participants are encouraged to continue other NHS cancer screening programmes and seek help for new or unusual symptoms. The primary objective is to demonstrate a statistically significant reduction in the incidence rate of stage III and IV cancers diagnosed in the intervention versus control arm 3–4 years after randomisation. NHS-Galleri will help determine the clinical utility of population screening with an MCED test.

## 1. Introduction

Cancer is a leading cause of death globally and the most common cause of death in high-income countries [1,2]. In 2019, there were more than 10 million deaths attributed to 29 cancer types combined across 204 countries and territories [3]. In England, approximately 140,000 deaths were caused by cancer [4], with lung and colorectal cancers being the leading causes of these deaths.

Cancer screening has the potential to reduce cancer mortality through earlier detection, which allows for more effective treatment [5]. There is a growing focus on the importance of detecting and diagnosing cancers earlier, including the promotion of early diagnosis by the World Health Organization [6]. Yet, in England in 2017, only 6% of all cancers were detected through National Health Service (NHS) screening programmes and 46% of all cancers with a known stage were detected at a late stage (III and IV) [7,8]. The COVID-19 pandemic has likely worsened the situation [9]. Given the overall cancer mortality burden in England, the NHS Long Term Plan has a key target to increase by 2028 the proportion of cancers diagnosed at an early stage (I and II) to 75% [10]. 

There are currently three national cancer screening programmes in England [11]. Colorectal, breast, and cervical cancer screening are offered to people aged 60–74, women aged 50–70, and women aged 25–64 years, respectively. Over 80% of cancer deaths in the UK are from cancers with no screening programmes [12]. Lung cancer accounts for almost 25% of all cancer deaths in the UK, yet it does not have a national screening programme at the time of writing [12]. The NHS is, however, piloting Targeted Lung Health Checks for those at high risk in selected areas of England, prior to a potential national programme [13]. Additional cost-effective cancer screening programmes with a favourable benefit-to-harm balance are therefore warranted.

Existing screening programmes are partially limited by low cancer prevalence for individual cancer types. With repeated, multimodal single-cancer screening, there is a high cumulative false-positive rate (the proportion of people without cancer who have received a positive screening result) [14]. For example, when existing screening tests for multiple different single-cancer types were implemented sequentially in the US as part of the Prostate, Lung, Colorectal, and Ovarian (PLCO) Cancer Screening Trial, the cumulative false-positive rate of 14 tests over 3 years was approximately 60% in men (serum prostate-specific antigen tests and digital rectal examinations for prostate cancer, chest radiographs for lung cancer, and flexible sigmoidoscopies for colorectal cancer) and 49% in women (serum tests to detect cancer antigen 125 and transvaginal sonograms for ovarian cancer, chest radiographs, and flexible sigmoidoscopies) [14]. Evidence also suggests that only a third of women in England aged 60–65 undergo all three recommended cancer screening programmes for which they are eligible (colorectal, breast, and cervical cancer screening), even though 90% participated in at least one of the three programmes [15]. 

In contrast to single-cancer screening approaches, a multi-cancer early detection (MCED) test takes advantage of the aggregated prevalence of multiple cancer types in a population and provides a single overall and relatively low false-positive rate. From the perspective of the individual participant, it is a simple blood test and therefore allows for flexibility with regard to administration settings. Ideally, an MCED test should (1) be able to detect clinically significant cancers in most organ systems, (2) have a low false-positive rate, and (3) have a relatively high positive predictive value in the target population [16]. A blood-based MCED test (Galleri^®^) was validated in the Circulating Cell-free Genome Atlas (CCGA) study, which had a case–control design (NCT02889978) [17]. This MCED test uses the methylation patterns of cell-free DNA (cfDNA; DNA released into the blood by apoptosis or necrosis of noncancerous and tumour cells) [18,19] to detect a shared cancer signal from more than 50 different cancer types (defined according to the eighth edition of the American Joint Committee on Cancer (AJCC) staging manual) and identify the cancer signal origin [17]. In this validation study, the MCED test had an overall specificity of 99.5% [17], which is considerably higher than common single-cancer screening tests [20,21,22,23,24,25,26,27]. Modelling of the potential performance of an earlier version of this MCED test (with plausible assumptions about tumour progression rates [‘fast scenario’]) in a representative US population suggested that, as part of an annual screening programme, it could reduce stage III and IV cancer incidence by 50% and thus reduce deaths within five years of a cancer diagnosis by 21% [28]. 

There are a number of US- and UK-based large, prospective, multicentre, studies evaluating MCED tests [29,30,31,32], including the interventional, single-arm, PATHFINDER (NCT04241796) study that enrolled 6662 asymptomatic participants in the US to assess clinical implementation, safety, and perceptions of the MCED test validated in the CCGA study [32,33,34]. PATHFINDER is one of the first trials to return MCED test results to healthcare providers and participants for intervention. 

The NHS-Galleri trial (ISRCTN91431511), a randomised controlled trial, is the first of its kind. It aims to establish, in persons not undergoing current investigation for suspected cancer (asymptomatic individuals), whether the MCED test can reduce the incidence rate of late-stage cancer in the intervention arm compared to the control arm. The results of this trial conducted within the NHS in England will help inform the use of the MCED test for population screening in high-income countries. Further follow-up using centrally linked databases will allow us to study the impact of the test on cancer-specific mortality. 

Here, we provide an overview of the NHS-Galleri trial, discuss factors that informed its design, and provide a rationale for several design features.

## 2. Materials and Methods

### 2.1. Trial Design

**Figure 1:**
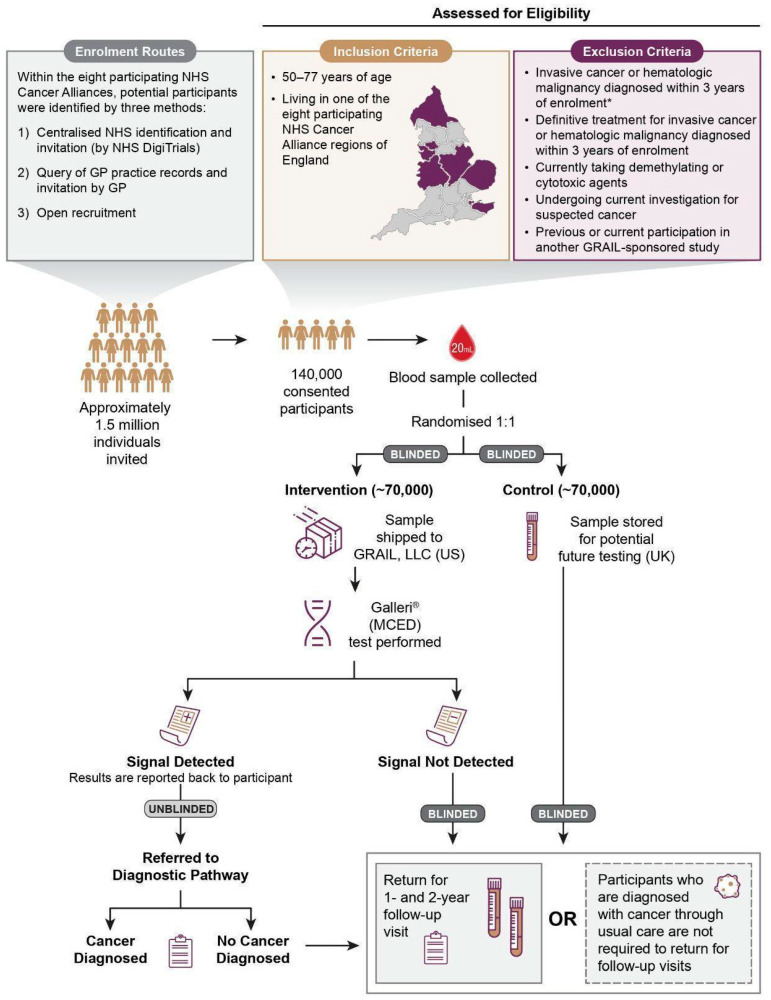
An overview of the NHS-Galleri trial. GP, general practitioner; MCED, multi-cancer early detection; NHS, National Health Service. * Individuals with non-melanoma skin cancer and individuals with prostate cancer whose only treatment is active surveillance are not excluded.

NHS-Galleri is a pragmatic, prospective, simple (individual-level), randomised controlled trial that has enrolled over 140,000 participants by inviting approximately 1.5 million people from the general population of England (Figure 1). The first participant was enrolled on 31 August 2021 and the last participant was enrolled on 16 July 2022. Participants and the study teams are blinded to study arm at the point of randomisation. Participants in the intervention arm with a cancer signal detected result are unblinded when they receive their test results. The clinician receiving the referral for diagnostic work-up of the patient receives the test report and is therefore non-blinded. The remaining participants stay blinded to minimise the risk of changes in health behaviours and encourage all participants to return for annual blood draws. Trial teams (with the exception of functions that require the information for sample management, testing and systems development) remain blinded throughout the study.

The trial received ethical approval from Wales Research Ethics Committee 1 (Ref: 21/WA/0141). The trial also received Health Research Authority (HRA) approval with support from the Confidentiality Advisory Group (Ref:21/CAG/0056), under Regulation 5 of the Health Service Regulations 2002 (‘Section 251 support’), for NHS Digital to send out invitation letters to eligible invitees to seek consent. The trial is being conducted in accordance with the protocol, the Declaration of Helsinki and Council for International Organizations of Medical Sciences International Ethical Guidelines, applicable International Conference on Harmonization/Good Clinical Practice Guidelines, and other applicable laws and regulations. 

### 2.2. Participants

Participant inclusion criteria were broad, and exclusion criteria were minimised in order to recruit a population representative of the intended use population, which will include people with other health conditions (see Figure 1). 

Potential participants were identified via three methods; the vast majority of participants through (1) centralised NHS identification and invitation by an NHS population database called NHS DigiTrials (a database that includes all individuals registered with an NHS General Practitioner (GP) in England, except for those who have exercised their right to the national data opt-out service, or opted out from receiving an invitation to the trial specifically via the NHS DigiTrials website), and a minority of participants through (2) query of GP practice records and invitation by a GP and (3) open enrolment of interested individuals who learned about the trial from specific targeted recruitment efforts in selected communities. 

**Figure 2:**
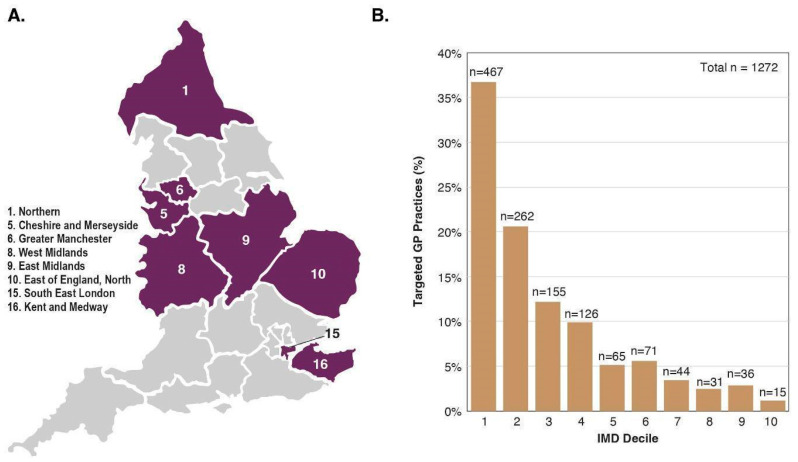
(**A**) Map of the eight participating NHS Cancer Alliances and (**B**) the IMD score distribution of targeted GP practices identified by NHS DigiTrials. (**A**) England has a total of 21 NHS Cancer Alliances. The regions shaded in purple are the eight participating NHS Cancer Alliances in the NHS-Galleri trial. (**B**) The IMD score distribution is from targeted GP practices identified by NHS DigiTrials. NHS DigiTrials invited eligible participants registered with these GP practices. The bar graph represents the percent of GP practices (y-axis) in each Cancer Alliance based on the IMD score (x-axis). IMD decile: 1 = most deprived, 10 = least deprived. GP, general practitioner; IMD, Index of Multiple Deprivation; NHS, National Health Service.

The trial aimed to enrol a representative sample of the population to ensure results are generalisable, to promote equity in healthcare and health research, and to minimise ‘healthy volunteer bias’ documented in prior screening studies [35,36]. We defined representativeness as including a reasonable absolute number of participants across sexes, neighbourhood-based socioeconomic groups, and major ethnic minority groups. We aimed to recruit at least 15,000 from each quintile of deprivation based on ranked neighbourhood groupings (Lower Layer Super Output Areas (LSOAs)s), at least 1500 from each major ethnic minority group (Black and Asian) and at least 12,000 in each five-year age group. We aimed to over-recruit older participants, in whom the burden of cancer is greatest. The eight participating Cancer Alliances (Figure 2A) were selected on the basis of a range of factors, including the level of socioeconomic deprivation and ethnic diversity, and relatively high cancer mortality and late-stage cancer diagnoses. Periodic characterisation and monitoring of the representativeness of enrolled participants compared to the general population aged 50–77 years was undertaken using participant-reported baseline data. Dynamic adjustment of invitation lists and targeted enrolment strategies were employed as needed. For example, we monitored the enrolment rates of participants from LSOAs by ranked deprivation index and dynamically adjusted invitation lists to target more participants from underrepresented groups [37]. Figure 2B shows the Index of Multiple Deprivation (IMD) score (1 = most deprived, 10 = least deprived) distribution of GP practices identified by NHS DigiTrials (from which eligible individuals registered at the GP practice were invited to attend), highlighting the targeted enrolment focus.

### 2.3. Randomisation and Masking

Following baseline blood draw at enrolment, participants were randomised 1:1 to one of the two arms of the trial: the intervention (blood sample analysed by the MCED test) or control (blood sample stored for research purposes (e.g., for secondary and exploratory analyses)). Only participants in the intervention arm with a cancer signal detected have results reported (unblinded) and are referred for diagnostic investigations and any necessary treatment. All other study participants remain blinded throughout the trial. The clinical report of a cancer signal detected result provides one or two predicted cancer signal origin(s). The first cancer signal origin provided by the MCED test is the most probable (top-predicted) cancer signal origin and the second, if appropriate, is the second most probable (second-predicted) cancer signal origin. Research nurses under the direction of The Cancer Research UK and King’s College London Cancer Prevention Trials Unit (CPTU) contact the participant by telephone to inform them of the result and refer the participant to an NHS urgent cancer referral pathway or rapid diagnostic pathway for confirmatory diagnostic assessment. The participant’s GP is informed of the result and referral.

The remaining participants in the intervention arm with a cancer signal not detected as a result, and those in the control arm, do not have any results reported back, and thus remain blinded as to whether they were randomised to the intervention or control arm to minimise the risk of changes in health behaviours and to encourage all participants to return for annual blood draws. They are informed by letter that their blood sample has been received and to await their next annual trial appointment for another blood draw. Participants will be encouraged to follow standard-of-care practices, including guideline-based cancer screening programmes, and report unusual or concerning symptoms to their GP. Except as required by specific roles, all trial personnel, trial sponsor employees, and other trial team staff remain blinded to accruing results. 

Participants in the intervention arm with a cancer signal detected result but no cancer diagnosed after confirmatory diagnostic workup will be invited for their next annual blood test. Although these individuals are unblinded, if they receive a result of cancer signal not detected at subsequent screening visits, they will be sent letters acknowledging the receipt of their sample as with the blinded participants.

### 2.4. Procedures

Blood is collected at three trial visits reflective of an annual screening approach, occurring at intervals of 1 year ± 6 weeks. These visits are referred to in this paper as baseline, second, and final trial visits, taking place at the beginning of the first, second and third rounds of screening (including 12-month follow up), respectively. A small number of participants (~1%) will have to be invited for redraws, either owing to sample or lab processing issues. In these instances, the visit interval will still be linked to the date of randomisation rather than redraw.

Participants who have a registered cancer diagnosis after randomisation, where the data on this have been made available to the trial team, will not be invited to return for subsequent trial visits, but will continue to be passively followed up via NHS datasets. All other participants without a registered cancer diagnosis will be invited to attend for a blood draw at one-year intervals. All phlebotomy appointments are carried out at mobile clinics. Accessibility requirements, such as wheelchair access, step-free access, disabled car parking, sighted or visual assistance, hearing assistance, and language interpretation are available at these clinics. 

At each blood collection appointment, 20 mL of peripheral blood is obtained by routine venipuncture, across two collection tubes. All blood samples are pre-processed. The resulting plasma samples are divided into two aliquots. The first aliquot of plasma is either analysed by the cfDNA-based targeted methylation MCED test that was evaluated in CCGA (intervention arm) or stored for research purposes (control arm) [17]. The second aliquot of plasma for participants in the intervention arm is to be used for testing if there are issues with processing the first. Any remaining samples are stored for later research.

For participants referred to an NHS cancer diagnostic pathway following a cancer signal detected as a result, no protocol-mandated confirmatory diagnostic procedures are set, rather, confirmatory diagnostic workup follows standardised clinical recommendations already in place to investigate symptoms that may indicate cancer at the site of the cancer signal origin(s) in the clinical report. An ‘interface document’ to support the diagnostic workup has been issued by NHS England. Decisions regarding which specific confirmatory diagnostic procedures should be performed are at the discretion of the physician. They base their decisions on medical judgement in accordance with the standard recommendations relevant to the cancer signal origin site(s) stated in the test report, and in consultation with the patient based on their medical history. Physicians are recommended to first exhaust confirmatory diagnostic procedures at the cancer signal origin site(s), and if no cancer is found, to consider a computed tomography (CT) chest–abdomen–pelvis scan, if not previously performed, before discharge. Details of diagnostic procedures undertaken will be gathered from routine NHS datasets, and all cancer diagnoses confirmed and described in detail through the National Cancer Registration and Analysis Service (NCRAS) [38].

All participants complete a questionnaire at the baseline visit and will be asked to complete follow-up questionnaires at the second and final visits (Table S1). The baseline visit questionnaire includes questions related to demographics, exposures and behaviours linked to increased cancer risk (e.g., smoking and alcohol use), personal and family history of cancer, participation in cancer screening programmes, medical history, and current medications. Additionally, the subset of participants with a cancer signal detected after the baseline visit will be sent three paper-based questionnaires over the year following the first blood draw. These questionnaires are designed to assess the Psychological Impact of the Galleri Test (sIG[n]al) as part of the secondary objectives. The questionnaires will include measures designed to assess the psychological consequences of receiving the test result (using the Spielberger State/Trait Anxiety Inventory-6 (STAI-6) and the Psychological Consequences of Screening Questionnaire (PCQ)) [39,40].

Instead of using electronic case report forms, outcomes data for the trial are routinely collected by linkage with central NHS data sources including, but not limited to, the NCRAS datasets [38], the Hospital Episode Statistics (HES) database [41], the Diagnostic Imaging Dataset (DID) [42], and Office of National Statistics (ONS) mortality data [43]. NCRAS is part of NHS Digital, which will oversee the data release of all trial data. NCRAS will complete the cancer registration process, which may take several months. GRAIL has funded additional Cancer Registration Officers employed directly by NCRAS. These employees support the cancer registration process for NHS-Galleri Trial participants by querying directly with hospital trusts providing care when there is missing information regarding a patient’s diagnosis or treatment. It is hoped this will improve the speed and completeness of the cancer registration process for trial participants. Personal data on consented individuals are being shared by the CPTU with NHS Digital to allow for identification and linkage. Personal data that have been pseudonymised are being shared by these organisations with the CPTU during the trial and for the duration of the passive follow-up to ensure high-quality and complete follow-up. Appropriate governance processes have been put in place to oversee the trial including a Trial Steering committee and an Independent Data Monitoring Committee.

### 2.5. Outcomes

The primary endpoint is the incidence rate of stage III and IV cancers adjusted by the follow-up time (up to 3–4 years after randomisation) in the intervention arm compared with the control arm. This will be assessed in the final analysis planned for 2025/2026.

Passive follow-up for cancer diagnoses will continue for all participants until one year after the final visit for the last participant, corresponding to a minimum of three and a maximum of four years follow-up after randomisation. Participants who do not attend the second or final visit will still be included in the final analysis. Primary, key secondary, and key exploratory objectives and endpoints are described in Table 1. 

### 2.6. Sample Size Calculation and Statistical Analysis

A microsimulation screening analysis model was used to determine (1) the likely effect size, by comparing the intervention and control arms, and (2) the sample size needed to secure a power of at least 90% at a significance level of 0.05 for a two-sided, two-sample comparison [44]. The microsimulation model simulates the natural histories of cancers and the interventions. In the model, participants were first simulated without MCED screening and subsequently randomised to the intervention and control arms, then MCED screening was simulated for participants in the intervention arm. The cancer diagnosis by standard-of-care and existing screening modalities for each individual in the simulation was modelled to match the empirical incidence of cancers diagnosed between 2012 and 2016 in England, as registered by NCRAS. Cancer type and stage for each individual were generated through a multinomial distribution with empirical probabilities. Cancer start time in current and prior stages was calculated by subtracting dwell time from cancer diagnosis time (with standard-of-care and existing screening modalities). Here, dwell time was defined as the length of time each cancer type would stay in each stage before progression to the next stage without intervention and was modelled as an exponential distribution. Participants in the intervention arm were simulated to be screened annually using the MCED test for three rounds, with 12 months follow-up after the third round of screening, assuming an 8% annual dropout rate. Sensitivities per cancer type per stage were based on CCGA substudy 2 [45]. We expect to observe earlier interception of cancers and stage shift towards earlier stages with MCED testing.

For the primary objective, the incidence rate of stage III and IV cancers detected by the MCED test plus standard-of-care modalities in the intervention arm will be compared with those detected by standard-of-care modalities only in the control arm, after an average 16–18 months of follow-up after the third round of screening. 

This objective will be evaluated using a fixed-sequence statistical strategy. First, we will evaluate for a statistically significant difference in a prespecified group of 12 cancer types: lung, head and neck, colorectal, pancreas, myeloma/plasma cell neoplasm, liver/bile duct, stomach, oesophagus, anus, lymphoma, ovary, and bladder [17]. If a significant reduction (*p* < 0.05) in incidence rates is found, we will then evaluate for a difference in all stageable cancer types (defined as invasive solid cancers (excluding basal cell carcinoma and squamous cell carcinoma of the skin) and haematological malignancies) other than prostate cancer; cancers not routinely staged (e.g., brain cancers and leukaemias) will be excluded. If the second evaluation also demonstrates a significant reduction in incidence rates, then all stageable cancers (including prostate) will be evaluated.

A two-sided test will be carried out to determine whether there is a statistically significant difference between the two arms in the incidence rate of stage III and IV cancers (*p <* 0.05). A point estimate of the incidence rate ratio of stage III and IV cancers (plus a 95% confidence interval) between the intervention and control arms will be reported.

The microsimulation model showed that, with a total sample size of 140,000 individuals (i.e., 70,000 per arm), under a range of assumptions for the cancer dwell time model, the study has a power of at least 90% to observe a significant difference in stage III and IV cancers (all sites combined) between the two arms at a significance level of 0.05 [44]. The microsimulation model predicts a relative reduction in stages III and IV cancers of approximately 20% after three rounds of MCED testing and one year of follow-up after the third round, with a cumulative incidence of stage III and IV cancers of approximately 1% in the control arm during this follow-up period.

## 3. Discussion

The NHS-Galleri trial is the first randomised controlled trial statistically powered to assess the clinical utility, including harms and benefits, of a blood-based MCED test for use in population cancer screening. The trial design reflects efforts to rapidly and robustly determine the efficacy of this potentially highly impactful screening programme to reduce the burden of late-stage cancer diagnoses. Conduct of the trial in a national health service allowing highly complete and uniform follow-up through routine linkage with NCRAS and other NHS data resources enables a pragmatic, large-scale trial with greater internal validity and less loss to follow-up than would be expected in comparable countries without national health services. Additionally, the absence of protocol-mandated diagnostic procedures in favour of an interface document guiding diagnoses through NHS pathways enables assessment of whether the intervention is implementation-ready. However, other trial design aspects (e.g., post-sample collection randomisation) are explanatory to test the efficacy of MCED screening in the intended population. 

It is important to first establish the efficacy of MCED screening in those screened, prior to measuring effectiveness at a population level of those invited for screening. Therefore, a post-sample collection randomisation design (see Figure 1) was chosen instead of the pragmatic Zelen’s design, which randomises participants prior to invitation, or after gauging interest in participating in a screening trial [46,47]. Post-sample collection randomisation ensures near 100% attendance at the initial (prevalent) screening visit, in contrast with Zelen’s design, where there is considerable attrition between randomisation and sample collection, negatively impacting the overall enrolment and statistical power of the trial design. If only 10% of those randomised to screening were screened, the study size would need to be over 100 times larger (i.e., 15 million people randomised). In this trial, the sample size of over 140,000 allows for: (1) loss to follow-up due to competing mortality; (2) diminished efficacy in those who are only screened once or twice (due to dropout); (3) healthy volunteer bias reducing the incidence of advanced-stage cancer in the control arm; and (4) reduced efficacy due to delayed diagnosis of cancer in individuals with a cancer signal detected in the intervention arm (the simulation assumes all cancers in individuals with a cancer signal detected at a given stage are diagnosed before progression to the next stage). This trial design has minimal risk of contamination due to participants in the control arm accessing the MCED via other routes, as it is not available in the UK outside of the trial setting at the time of writing (unlike the United States). Furthermore, this design enables retrospective analysis of samples collected from the control arm to determine what the MCED test result would have been if these participants had been screened. 

The NHS-Galleri trial is intended to influence considerations for a national rollout of an MCED-based screening programme, both in the UK and in countries with comparable demographic characteristics, cancer burden, and healthcare systems. Following the prevalent (initial) screening round, more cancers will be screen-detected, and two incident (subsequent) screening rounds will enable better estimation of the impact of regular screening, including the cumulative test performance (e.g., sensitivity and specificity) of sequential annual screening. Undertaking three screening rounds ensures trial results are not unduly influenced by short-term changes in the prevalent pool of cancers or cancer service delivery, for example as a potential result of the COVID-19 pandemic [9,48,49]. 

Another critical design choice in cancer screening trials involves the timing of endpoint assessment. Insufficient follow-up time may not capture benefits of screening. Our microsimulation model suggests that it takes three rounds of annual MCED testing and one year of follow-up after the last round of MCED testing to ensure sufficient statistical power for the comparison of late-stage cancer incidence [44]. 

The key goal of cancer screening interventions is to reduce mortality [11] and effective interventions should be implemented as quickly as possible to this end. However, there is debate regarding which trial endpoints sufficiently balance these competing needs. Here, we chose reduction in late-stage cancers over other options, including cancer-specific or all-cause mortality, which both require larger populations and longer follow-up times. All-cause mortality also arguably lacks specificity and fails to distinguish between benefits and harms [50,51]. This trial was not primarily powered to assess mortality reduction, as it would likely require at least 6 years follow-up. However, there is good evidence that reduction in late-stage cancers translates into mortality benefits [52,53]. Therefore, it is appropriate to plan for widespread implementation of MCED population screening if the results show a reduction in the occurrence of late-stage cancers. This could be conducted while generating further data on cancer-specific mortality, via extended follow-up and retrospective testing of samples from individuals in the control group who die of cancer. 

For most cancer types, the strong prognostic significance of stage at diagnosis is well established [54]. However, there are still three key challenges regarding the extent to which late-stage reductions translate into mortality reduction. First, in some single-cancer screening trials, late-stage reduction did not translate into mortality benefit. For example, in the UK Collaborative Trial of Ovarian Cancer Screening (UKCTOCS) trial of blood cancer antigen CA125 measurements and transvaginal ultrasound scans, the observed nonsignificant 10.2% reduction in late-stage (III and IV) ovarian cancer incidence in the screening versus no screening cohort (*p* = 0.084) was substantially attenuated when it came to disease-specific mortality (nonsignificant 4.4% reduction) with a median of 15 years of follow-up [36], which led some to conclude that mortality endpoints are necessary in screening trials. The null result may have been due to a stage shift of insufficient magnitude, dilution effects of long follow-up, or simply that the lead time provided by the screening used in that trial was insufficient to produce a reduction in late-stage cancers (the reduction was not significant even without adjustment for a secondary endpoint). Other studies have reached opposing conclusions. For example, a meta-analysis of breast cancer trials demonstrated that observed reductions in non-localised (≥20 mm in size, ≥stage II, or node-positive) breast cancer diagnoses were strongly correlated with observed reductions in cancer mortality [52,53]. There is likely a difference between cancer types in the degree to which stage shift produces mortality benefits. From the microsimulation model used to design this trial, we anticipate an overall reduction in late-stage (III and IV) cancer incidence of 16–29%, and earlier modelling suggested that the reduction in cancer mortality will be approximately 40–45% of the reduction in late-stage disease [28]; thus, realising a mortality benefit is more likely.

The second concern for not using cancer-specific mortality as the primary endpoint is that while some prior screening interventions have successfully demonstrated an increase in the number of cancers diagnosed at an early stage, these additional cancers may reflect overdiagnosis [55]. However, in this trial, framing the endpoint in terms of late-stage reduction rather than increased early-stage diagnoses safeguards against a positive trial result on the basis of increased indolent early stage diagnoses. Moreover, the MCED signal is based upon circulating tumour fraction (cTF; the proportion of circulating tumour DNA (ctDNA) in cfDNA), which appears to reflect important clinical and prognostic information [56]. Clinical validation of the MCED test in the third CCGA substudy demonstrated increased overall sensitivity by stage and higher overall sensitivity in the 12 prespecified, clinically significant cancer types [17]. The test also has lower sensitivity for cancers which are more commonly overdiagnosed (e.g., screen-detected breast, prostate, thyroid) [55]. Thus, the scope for overdiagnosis is also limited by the basis of the MCED test signal, and the subsequent test performance characteristics. 

The final concern is that earlier diagnosis may not ultimately alter the disease course, and participants with cfDNA-detectable tumours still die at the same point in the absence of this intervention. Cell-free DNA positive (cfDNA^+^) lung tumours have been found to be enriched for subclinical micrometastases [57], and cTF is correlated with clinical biomarkers of poor prognosis [56]. Data from the second CCGA substudy in participants with clinically diagnosed cancers demonstrated that tumours that were cfDNA+ had worse survival than site- and stage-matched tumours that were cfDNA-by MCED testing [56]. However, the survival of participants with cfDNA^+^ cancers in CCGA was comparable to site- and stage-matched data from the Surveillance Epidemiology and End Results programme (SEER) [58]. Other data have also demonstrated that some cfDNA^+^ tumours remain free from recurrence after treatment [59]. Detecting and downstaging the worst-prognosis tumours necessitates better understanding of the appropriate treatments for these tumours, which may involve either escalated systemic anti-cancer therapy regimens currently in use, or the development of new treatments. The benefit-to-harm ratio of MCED screening may increase over time as knowledge of optimal treatment approaches for cfDNA-shedding tumours improves. cTF could feasibly complement clinical staging as a biomarker to influence treatment decisions. 

Owing to this concern regarding the prognostic implications of cfDNA detectability, and given that the ultimate goal of cancer screening is to reduce cancer-specific mortality, we designed this trial to understand the impact of MCED screening on cancer-specific mortality. As previously mentioned, the trial will be under powered to directly compare cancer-specific mortality between arms. Instead, we can test the stored samples from controls who die from cancer, and compare the number of participants who both died from cancer and had a cancer signal detected result between arms. This nested analysis is to test whether having a cancer signal detected result in the intervention arm prevents (some of) those individuals from dying from cancer [60]. This endpoint would not be diluted in either arm by cancer deaths in individuals whose cancers were (or would have been) missed by the MCED test, increasing the power.

The NHS-Galleri trial design has limitations. We will only evaluate MCED population screening as an annual test in participants aged 50–80 years. The trial is not powered to evaluate any of the following: the benefit of screening separately in specific socioeconomic, age, or ethnic subgroups; the clinical utility for each individual cancer type detectable using this MCED test; or the relative benefits of different screening schedules, such as biannual or biennial screening. The trial is also not designed to answer whether MCED screening could replace or change the frequency of other current single-cancer screening programmes. The pragmatic design aspect of having an interface document to help guide diagnostic procedures rather than protocol-mandated confirmatory diagnostic procedures may confound the impact of MCED testing. For example, there may be variability in the point at which a clinician decides to end confirmatory diagnostic workup following negative diagnostic test results at up to two cancer signal origin sites. However, it allows for clinical judgement and appropriate use of local diagnostic resources within the NHS. This was chosen to better assess whether or not the MCED test result can be incorporated into routine clinical care in the NHS. The extensive routine datasets within the single system of the NHS do not capture the reasons for undertaking medical procedures or hospital events, which is problematic for the healthcare resource utilisation and safety endpoints. In cases where reasons are ambiguous, causality between cancer signal detection and subsequent confirmatory diagnostic procedures along with any resulting complications will be inferred based on temporal association only. The difference between the trial arms will provide insight into the overall impact of MCED screening on healthcare resource utilisation. Despite this limitation, the use of these datasets is a pragmatic choice that will allow for passive follow-up and enable the scale of the trial, while ensuring comprehensive population coverage (hospitals are mandated to submit data to NCRAS on NHS-funded activity, which is estimated to be 98–99% of the hospital activity in England [41]). Screening trials such as this cannot accurately estimate the likely uptake of a national screening committee-recommended screening programme. Participation in a trial involves having blood taken, which may or may not be tested using a screening test for which clinical utility has not yet been demonstrated. Further research is needed to investigate this issue and potential screening implementation barriers. Despite this inherent limitation, we can evaluate the demographics and characteristics (e.g., age, gender, ethnicity, and socioeconomic group) of individuals who are invited to attend and participants who enrol in the trial. These data may provide preliminary insights into the uptake of the MCED test in these subgroups. 

The limitations of this trial design are balanced by its strengths. Most importantly, the trial will yield results within about 4–5 years of the first person randomised. In the context of previous cancer screening trials, in which it took 10–15 years to generate results, this is incredibly rapid [61,62]. Conduct of the trial in a national health service ensures systematic and complete follow-up of all participants for key cancer outcomes regardless of residential or occupational changes during the course of the study as might occur in a more fragmented health system. To ensure trial results could be generalised to the intended-use population, we did not exclude participants on the basis of having health conditions/comorbidities. There will be no lead- or length-time bias, as the time of events will be measured from randomisation (first blood draw) rather than from cancer diagnosis. Simple rather than cluster randomisation maximises representativeness and minimises bias and the required sample size. Moreover, participant enrolment was evaluated weekly for representativeness to the target population with respect to ethnicity and socioeconomic group to better address health equity and minimise healthy volunteer bias. Dynamic adjustment ensured more invitations were sent to people from underrepresented groups. A range of methods were applied to support enrolment of a representative trial population, including translation services, and locating mobile clinics in areas of higher socioeconomic deprivation. We aim to maximise access throughout the trial by locating the mobile clinics at supermarket car parks, or sites with good transport links. Mobile clinics were favoured over more traditional settings for phlebotomy, such as GP practices or hospitals, to avoid overburdening these already stretched services. We also hope that placing these mobile clinics in more familiar and accessible locations may reduce the barriers some sociodemographic groups experience with accessing healthcare. Participants in the intervention arm who have a cancer signal not detected result and all participants in the control arm will remain blinded to help minimise confounding changes in health behaviours. This will enable the most accurate estimate of the clinical utility of the MCED test and reduce the risk of control arm participants not returning for follow-up trial visits. Importantly, the inclusion of patient-centric endpoints, including assessment of the psychological impact of receiving a cancer signal detected result, will reflect the whole cancer-related experience in participants with a cancer signal detected. The Economic Evaluation Policy Research Unit (EEPRU) was appointed by NHS England to lead an independent economic evaluation of the MCED test, which will provide insight into the impact of MCED screening for the financial sustainability of the NHS. Lastly, the trial will measure potential harms that are inherent to all cancer screening programmes, including psychological harms, potential overdiagnosis and corresponding overtreatment, and complications of confirmatory diagnostic procedures following a cancer signal detected result.

## 4. Conclusions

In summary, the NHS-Galleri trial is designed to assess the clinical utility of a blood-based MCED test alongside the current standard of care. The results of the trial will help inform decisions about whether the MCED test should be used in a population screening setting. Future research can implement design features of this trial to advance early cancer detection through screening. 

## Figures and Tables

**Table 1:** Primary, key secondary, and key exploratory objectives and endpoints.

Objective/Endpoint		First Screening Round *	Second Screening Round *	Third Screening Round *	Three Screening Rounds Aggregated *	3–4 Years after Randomisation ^†^	3 Years after Final Visit	6 Years after Final Visit
Primary objective/endpoint	Demonstrate a significant reduction in the incidence rate of stage III and IV cancers diagnosed in the intervention arm compared with the control arm					X		
Key secondary objectives/endpoints	Demonstrate a significant reduction in the incidence rate of stage IV cancers diagnosed in the intervention arm compared with the control arm (excluding cancers identified by the test performed at the second visit)	X						
	Evaluate the MCED test performance (overall sensitivity, specificity, PPV, NPV, and cancer signal origin accuracy) in the intervention arm	X	X	X	X			
	Evaluate the safety, including harms, in the intervention arm among participants with a cancer signal detected result by assessing the number of complications and deaths resulting from confirmatory diagnostic procedures, estimated radiation exposure per participant due to test result-directed evaluations, and participant-reported psychological impact among participants with a cancer signal detected result	X	X	X	X	X		
	Assess the impact of the use of the MCED test across three annual timepoints on healthcare resource utilisation for cancer diagnosis and treatment, by measuring the number of follow-up procedures and number of invasive procedures needed to achieve diagnostic resolution among participants with a cancer signal detected result, and the number and type(s) of medical encounters and cancer-specific confirmatory diagnostic and treatment procedures among participants with a cancer signal detected result				X			
	Compare cancer-specific mortality in the intervention and control arms using a retrospective nested analysis					X (for 12 prespecified cancer types ^‡^)	X	X
	Assess the potential impact of overdiagnosis by studying the excess in cancers diagnosed after a baseline cancer signal detected result in the intervention arm compared with the control arm (retrospectively testing baseline samples from all participants diagnosed with cancer in the control arm)					X		
Key exploratory objectives	Retrospectively test the participants in the control arm who were diagnosed with a cancer of unknown primary and report the cancer signal origin detected by the MCED test					X		
	Assess the primary and secondary objectives in clinically meaningful subsets (e.g., by age, gender, ethnicity, socio-economic groups, risk factors at enrolment, prior cancer history at enrolment)					X		
	Assess the potential for avoidance/postponement of cancer death by comparing the cancer-specific mortality rates among participants with a baseline cancer signal detected result in the intervention arm versus the control arm (retrospectively testing baseline samples in the control arm)					X		
	Assess any potential impact of a baseline cancer signal detected result on non-cancer and all-cause mortality by comparing cancer signal detected non-cancer deaths and cancer signal detected all-cause mortality in the intervention arm compared with the control arm (retrospectively testing baseline samples in the control arm)					X		

MCED, multi-cancer early detection; NPV, negative predictive value; PPV, positive predictive value. * Including the visit at which blood is collected, and approximately 12 months of follow-up. ^†^ Enrolment will take approximately 1 year from the first participant to the last participant. Follow-up for cancer diagnoses will continue for all participants until 1 year after the final visit for the last participant. This will provide an average of 3–4 years of follow-up after randomisation. ^‡^ Lung, head and neck, colorectal, pancreas, myeloma/plasma cell neoplasm, liver/bile duct, stomach, oesophagus, anus, lymphoma, ovary, and bladder.

## Data Availability

Data sharing is not applicable to this article as the article is a study design of a trial in progress.

## Supplementary Materials

The following supporting information can be downloaded at: https://www.mdpi.com/article/10.3390/cancers14194818/s1, Table S1: Participant Questionnaire.
