# Supplementary material for: Cell-Free DNA–Based Multi-Cancer Early Detection Test in an Asymptomatic Screening Population (NHS-Galleri): Design of a Pragmatic, Prospective Randomised Controlled Trial
Source: Cancers (Basel). 2022 Oct 1;14(19):4818. doi: 10.3390/cancers14194818 (PMC9564213; doi:10.3390/cancers14194818)
Supplement: Supplementary file 1 [file cancers-14-04818-s001.zip › cancers-1908651-supplementary.pdf]

Supplementary Materials

Table S1. Participant questionnaire.

**Question number**	**Questions**	**Electronic answer choices**
**SECTION: Basic information**		
1	Which of these categories best describes your ethnic group?	White English, Welsh, Scottish, Northern Irish, or British<br>White Irish<br>Gypsy or Irish Traveller<br>Other White<br>White and Black Caribbean<br>White and Black African<br>White and Asian<br>Other Mixed<br>Indian<br>Pakistani<br>Bangladeshi<br>Chinese<br>Other Asian<br>Black Caribbean<br>Black African<br>Other Black<br>Arab<br>Any other ethnic group
2	Were you born in the United Kingdom?	Yes<br>No<br>I decline to answer this question
3	At what age did you begin living in the United Kingdom?	<<Open-ended question>>

4	What is your current marital status?	Single, never married<br>Married, in a civil partnership or living together<br>Divorced or separated<br>Widowed, not remarried<br>I decline to answer this question
5	What is the highest level of education you have achieved?	Finished school at or before the age of fifteen<br>Completed CSEs, O-levels or equivalent<br>Completed A-levels or equivalent<br>Completed further education but not a degree<br>Completed a Bachelor’s degree or equivalent<br>Completed a further degree, eg, masters or PhD etc
6	What is your current weight? You can report this in kilograms, or stones and pounds.	<<Open-ended question>>
7	What is your current height? You can report this in centimetres, or feet and inches.	<<Open-ended question>>
**SECTION: Smoking, alcohol use, and other exposure**		
8	Have you smoked at least 100 cigarettes in your lifetime?	Yes<br>No

9	Do you currently smoke cigarettes regularly?	Yes<br>No
10	At what age did you start smoking cigarettes regularly?	<<Open-ended question>>
11	At what age did you stop smoking cigarettes?	<<Open-ended question>>
12	How many cigarettes do or did you<br>smoke per day on average for the<br>majority of your time as a smoker?	<<Open-ended question>>
13	Have you ever had a drink containing alcohol?	Yes<br>No<br>I decline to answer this question
14	Do you currently drink alcohol?	Yes<br>No<br>I decline to answer this question
15	During the time when you drank alcohol, HOW OFTEN did you drink alcoholic drinks?	Less than once a week<br>Once a week<br>Twice a week<br>3 times a week<br>4 times a week<br>5 times a week<br>6 times a week<br>Every day<br>I decline to answer this question

16	HOW MANY drinks did you have on days when you drank alcohol?	Fewer than one drink per day<br>1 drink per day<br>2 drinks per day<br>3 drinks per day<br>4 drinks per day<br>5 or more drinks per day<br>I decline to answer this question
17	In your home or at work, are you aware of having been in contact with asbestos, coal dust, wood dust, or other mineral, rubber, or metal dusts without using protective equipment?	Yes, frequently<br>Yes, rarely<br>No<br>I don't know<br>I decline to answer this question
**SECTION: Personal and family medical history**		
18	Are you worried that you might have cancer?	Yes<br>No
19	Do you have any signs or symptoms that make you think that you might have cancer?	Yes<br>No

20	Has your doctor ever told you that you have any of the following chronic health/medical conditions?	Barrett's oesophagus<br>Chronic bowel disease (eg, Crohn's disease or ulcerative colitis)<br>Chronic liver disease (eg, chronic hepatitis infection, fatty liver disease)<br>Diabetes<br>Chronic pancreas disease (eg, pancreatitis)<br>systemic lupus erythematosus (SLE, "lupus")<br>Other health conditions (eg, multiple sclerosis, stomach ulcers)<br>None of the above
21	Have any of your relatives ever been diagnosed with cancer?	Yes<br>No
Questions related to Question #21	Which of the following relatives of yours have been diagnosed with cancer?	Mother<br>Father<br>Grandmother<br>Grandfather<br>Sister<br>Brother<br>Aunt<br>Uncle<br>Nephew<br>Niece<br>Son<br>Daughter<br>Other relative

	Which cancer was this relative diagnosed with?	Anus or anal canal<br>Appendix<br>Bladder<br>Brain and other nervous system<br>Breast<br>Cervix<br>Colon or rectum<br>Endometrium (uterus)<br>Gallbladder<br>Head and neck<br>Kidney<br>Lymphoma (Hodgkin and Non-Hodgkin)<br>Leukaemia<br>Liver, bile duct<br>Lung<br>Melanoma of the skin<br>Neuroendocrine<br>Oesophagus<br>Ovary<br>Pancreas<br>Prostate<br>Sarcoma<br>Small intestine<br>Stomach<br>Thyroid<br>Vagina or Vulva<br>I don't know<br>Other
	Specify other cancer	<<Open-ended question>>
	Do you know how old this relative was when diagnosed?	Yes<br>No
	Age at diagnosis	<<Open-ended question>>

22	To screen for cancer, the NHS invites men and women to have screening tests (for breast, cervical, or bowel cancer).<br>Which of the following best describes you?	I have never heard of cancer screening<br>I have heard of cancer screening but have never been invited<br>I have been invited for cancer screening but have never done it<br>I have been invited for cancer screening but have not done it every time I was invited<br>I have been invited for cancer screening and have done it every time I was invited<br>I don't know<br>I decline to answer this question
**SECTION: Women's reproductive history**		
23	In the next question(s) we will ask you about having children. The information you tell us will help us to understand more about the people we hope the test will help. If you don't want to give information about your past pregnancies, please choose I decline to answer this question.<br>Have you ever been pregnant?	Yes<br>No<br>I decline to answer this question
24	How many of your pregnancies resulted in the birth of a live child?	<<Open-ended question>>

25	How old were you when your first child was born?	<<Open-ended question>>
26	Have you ever taken hormone therapy (prescribed for women for menopausal symptoms, eg, oestrogen pills, patches or vaginal cream gel or ring, or progestin)?	Yes, and I am currently taking it<br>Yes, but I do not take it currently<br>No, never<br>I don't know<br>I decline to answer this question
27	How many years total have you used hormone therapy for menopausal symptoms? If you stopped and started, please add up the total time.	Less than one year<br>1 year<br>2 years<br>3 years<br>4 years<br>5 years<br>6 years<br>More than 6 years<br>I don't know<br>I decline to answer this question
**SECTION: Men's health**		
28	Men can have a blood test, called a PSA test, which looks for a possible indicator of prostate cancer. Have you ever had a PSA test?	Yes<br>No<br>I don't know<br>I decline to answer this question
29	When did you last have a PSA test?	Within the last five years<br>More than five years ago<br>I don't know<br>I decline to answer this question

SECTION: Your feelings about your health		
30	Please select the ONE box that best describes your health TODAY.<br>- MOBILITY	I have no problems in walking about<br>I have slight problems in walking about<br>I have moderate problems in walking about<br>I have severe problems in walking about<br>I am unable to walk about
31	Please select the ONE box that best describes your health TODAY.<br>- SELF-CARE	I have no problems washing or dressing myself<br>I have slight problems washing or dressing myself<br>I have moderate problems washing or dressing myself<br>I have severe problems washing or dressing myself<br>I am unable to wash or dress myself
32	Please select the ONE box that best describes your health TODAY.<br>- USUAL ACTIVITIES (eg, work, study, housework, family or leisure activities)	I have no problems doing my usual activities<br>I have slight problems doing my usual activities<br>I have moderate problems doing my usual activities<br>I have severe problems doing my usual activities<br>I am unable to do my usual activities
33	Please select the ONE box that best describes your health TODAY.<br>- PAIN / DISCOMFORT	I have no pain or discomfort<br>I have slight pain or discomfort<br>I have moderate pain or discomfort<br>I have severe pain or discomfort<br>I have extreme pain or discomfort

34	Please select the ONE box that best describes your health TODAY.<br>- ANXIETY / DEPRESSION	I am not anxious or depressed<br>I am slightly anxious or depressed<br>I am moderately anxious or depressed<br>I am severely anxious or depressed<br>I am extremely anxious or depressed
35	We would like to know how good or bad your health is TODAY. You will see a scale numbered from 0 to 100. 100 means the best health you can imagine. 0 means the worst health you can imagine. Please indicate on the scale how your health is TODAY.	0 to 100
